# Is high salt intake inducing obesity via production of cortisol? A novel working hypothesis and pilot study

**DOI:** 10.1007/s00394-024-03354-6

**Published:** 2024-02-26

**Authors:** Anthony Nowell, Susan J. Torres, Sarah J. Hall, Michelle A. Keske, David J. Torpy, Lewan Parker, Andrew C. Betik, Anne I. Turner

**Affiliations:** 1 Institute for Physical Activity and Nutrition, Deakin University, Geelong, VIC Australia; 2 Endocrine and Metabolic Unit, Royal Adelaide Hospital, Adelaide, SA Australia

**Keywords:** Salt, Sodium, Salt intake, Cortisol, ACTH, Obesity

## Abstract

**Purpose:**

Evidence is growing that high salt intake is an independent risk factor for obesity, but the mechanisms are unknown. Our novel working hypothesis is that high salt intake drives cortisol production, which in turn, drives obesity. The current study aimed to demonstrate an acute cortisol response following a single high salt meal.

**Methods:**

Eight participants (age 30.5 ± 9.8 years [mean ± SD], 50% female), consumed high salt (3.82 g; 1529 mg sodium) and low salt (0.02 g; 9 mg sodium) meals in a randomized cross-over design.

**Results:**

Urinary and salivary cortisol and plasma adrenocorticotropic hormone (ACTH) demonstrated order effects. When high salt was given second, there was a peak above baseline for urinary cortisol (26.3%), salivary cortisol (9.4%) and plasma ACTH (4.1%) followed by a significant decline in each hormone (treatment*time, F[9, 18] = 2.641, p = 0.038, partial η^2^ = 0.569; treatment*time, F[12, 24] = 2.668, p = 0.020, partial η^2^ = 0.572; treatment*time, F[12, 24] = 2.580, p = 0.023, partial η^2^ = 0.563, respectively), but not when high salt was given first (p > 0.05 for all).

**Conclusion:**

These intriguing findings provide partial support for our hypothesis and support a need for further research to elucidate the role of high salt intake in cortisol production and, in turn, in the aetiology of obesity.

**Trial registration number:**

ACTRN12623000490673; date of registration 12/05/2023; retrospectively registered.

**Supplementary Information:**

The online version contains supplementary material available at 10.1007/s00394-024-03354-6.

## Introduction

Despite World Health Organization recommendations to limit salt intake to 5g (2000 mg sodium) or less per day [1], global average salt intake remains stubbornly high at 10 g (3950 mg sodium) per day with some variation by region [2]. Links between high salt intake and increased risk of cardiovascular disease and stroke are well documented [3].

A growing body of evidence shows high salt intake may be an independent risk factor for obesity [4–6]. A recent meta-analysis showed BMI, weight category and waist circumference remained positively associated with sodium intake with consistent and unchanged effect size after adjustment for energy intake [6]. An additional 1 g/day of salt was associated with 0.32 kg/m^2^ higher BMI [6]. An earlier cross-sectional study of 785 adults showed 1 g/day additional salt intake was related to 26% higher risk of obesity, after adjustment for factors including energy intake [5]. Higher salt intake was also significantly related to higher body fat mass independent of energy intake [5]. A 6-year longitudinal study showed higher salt intake was associated with greater increases in body fat mass and a reduction in fat free mass, after adjusting for total energy intake and change in body weight [4]. These findings support the involvement of high salt intake in the development of obesity, rather than high salt intake simply being indicative of a poor diet overall. Potential mechanisms by which high salt intake may contribute to obesity are unknown but deserve investigation.

A parallel body of evidence is increasingly reporting that high salt intake is related to higher urinary excretion of the metabolic and stress hormone cortisol [7–13]. Cross-sectional studies have shown a positive association between 24-h urinary sodium excretion (the gold standard for measuring salt intake [14]), and urinary cortisol [7, 8]. Interestingly, when plasma/serum cortisol rather than urinary cortisol was measured, no association with urinary sodium was found [7, 15], highlighting the need to investigate urinary cortisol in this context. Feeding studies provide further support for salt-induced cortisol production with dietary salt loading for 5–7 days increasing 24-h urinary cortisol [9–12] and dietary salt restriction for 2 and 7 days reducing 24-h urinary cortisol [13].

Links between high cortisol levels and obesity have been well known for many decades amongst clinical endocrinologists [16, 17] and researchers [18–20]. The ‘abdominous body had the appearance of a full-term pregnancy’ reported the physical examination of an early twentieth century Cushing’s patient [16], in which a pituitary tumour leads to excessive cortisol exposure [17]. Increases in endogenous and exogenous glucocorticoids in an experimental rat model increased mesenteric fat accumulation [18], and long-term cortisol exposure in humans (measured objectively by hair cortisol concentrations) was associated with higher weight, BMI and waist circumference and persistence of obesity [19]. Even the common practice of using prescribed corticosteroid treatments is related to higher BMI and waist circumference [20]. Consequently, findings brought together from these disparate disciplines introduce cortisol as a potential mechanism driving the relationship between high salt intake and obesity.

Collectively, these findings reported above bring us to our novel working hypothesis:

High salt intake drives cortisol production, and in turn, increased cortisol production drives obesity.

Cortisol can be produced either via the hypothalamic-pituitary adrenal (HPA) axis [21] or via 11β-hydroxysteroid dehydrogenase type 1 (11βHSD-1) conversion of its inert metabolite cortisone to cortisol in the periphery [22]. The discrepancy (described above) in which salt intake is related to urinary cortisol but not plasma/serum cortisol may provide insight to the source of salt-induced cortisol production. A high-salt diet in rats was shown to increase 11βHSD-1 activity and glucocorticoid concentrations in visceral adipose tissue [23]. If salt intake induces cortisol production in visceral adipose tissue in humans, the newly produced cortisol may be cleared rapidly by the kidney upon entry into the systemic circulation, consistent with findings above showing a lack of cortisol measured in plasma/serum. Cortisol produced within visceral adipose tissue is well situated to influence visceral fat accumulation. Cortisol production via conversion from cortisone would be expected to occur in the absence of ACTH production—ACTH is essential for production of cortisol via the HPA-axis, but not for conversion from cortisone. Consequently, measurement of ACTH may help elucidate the origin of cortisol production induced by high salt intake.

The current study sought to shed light on the lesser understood first part of our novel working hypothesis (i.e. that high salt intake drives cortisol production). While studies to date show an association between high dietary salt-intake over several days and 24-h urinary cortisol, no studies have investigated the acute relationship to obtain support for a cause-and-effect timeframe. The primary aim of our study was to demonstrate salt-induced cortisol production in an acute setting. Our secondary aim was to demonstrate this cortisol production in the absence of an ACTH response—if cortisol is produced locally in visceral adipose tissue via conversion from cortisone, activation of the HPA axis is not required/involved. We hypothesized that urinary cortisol but not plasma ACTH will increase following a single high-salt meal. To further characterize the role of high salt intake, we measured salivary cortisol, heart rate (HR), systolic blood pressure (SBP), diastolic blood pressure (DBP), and blood glucose.

## Materials and methods

### Theoretical approach

We have taken a multidisciplinary approach to test the first part of our novel working hypothesis—that high salt intake drives cortisol production. To achieve this outcome, we have integrated experimental methods previously used in nutrition research for salt intake studies [8, 24, 25] with experimental methods previously used in stress/psychoneuroendocrinology research for measuring stimulus-induced reactivity of the hypothalamo-pituitary adrenal axis and sympatho-adrenal medullary system [26–29]. Through this combination of methods from disparate disciplines, we were able to investigate a new and relatively unexplored physiological pathway that may shed light on the aetiology of obesity.

### Participants

Eligible participants were male or female, 18–50 years of age and not taking medication for blood pressure or diabetes. After responding to ads, recruits were excluded during a phone interview if they reported any diagnosis with Cushing’s syndrome, any stress or anxiety disorder, depression, any diseases of the adrenal gland, Type 2 diabetes, heart disease, high cholesterol, stroke or cancer or use of medication known to affect cortisol levels [26, 28]. Pregnant or breast-feeding females were excluded, as were participants who, at the start of their first visit to our Clinical Research Facility, recorded resting blood pressure > 160/90 mmHg or body mass index (BMI) ≥ 30 kg/m^2^ [26, 28]. Deakin University Human Research Ethics Committee approved all procedures (Project number 2018-384) and all participants provided written informed consent.

### Study design

**Fig. 1:**
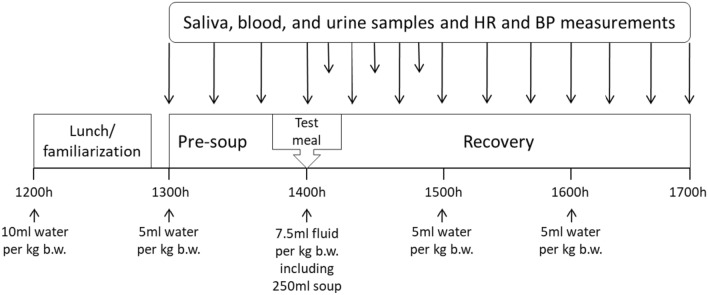
Study design on participants' second and third visits. Saliva, blood, and urine samples and HR and BP measurements were collected every 20 min (long arrows) from 1300 to 1700. Additional saliva and blood (but not urine) samples and HR and BP measurements were collected at 10-min intervals (short arrows) during the first hour after ingestion of the test meals. *HR* heart rate, *BP* blood pressure, *b.w.* body weight

Eight participants (n = 4 females and 4 males) were included in a randomized cross-over design. Additional measures were collected from eligible participants at the initial visit including waist and hip circumference and body composition (whole-body dual-energy X-ray absorptiometry, DEXA). A fasting blood sample was collected for cardiometabolic risk markers (fasting serum glucose, insulin, C-reactive protein, high-density lipoprotein, low-density lipoprotein, triglycerides, and total cholesterol). The main study took place over visits two and three to our research facility, which took place 6 or more days apart from each other (median = 8 days, range 6–21 days). This wash-out period was similar between those who had the high salt meal first and those who had the high salt meal second (median = 7 and 9 days, range 6–21 and 7–15 days, respectively). To mitigate any potential influence of the diurnal pattern of cortisol [30], we standardised the time of day for the experimental testing for all participants. Upon arrival at 1200 h, participants consumed a low protein meal, an intravenous cannula was inserted into the arm and participants were familiarized with the sampling procedures (Fig. 1). At 1400 h, participants consumed either a high salt meal (3.82 g salt, 1529 mg sodium) or a low salt meal (0.02 g salt, 9 mg sodium), in randomized order. Saliva, blood and urine samples and HR, SBP and DBP measurements were collected every 20 min from 1300 h until 1700 h. Additional saliva and blood (but not urine) samples and cardiovascular measurements were taken at 10-min intervals between 1400 and 1500 h. To maintain urine flow rate, water was consumed at 1200 h (10 mL per kg body weight [b.w.]) and at 1300, 1500 and 1600 h (5 mL per kg b.w.) and fluid containing water and the test meal was consumed at 1400 h (7.5 mL per kg b.w. including 250 mL test meal, Fig. 1). All participant visits to our study facility took place from May–September 2019.

### Anthropometry

Height, weight and waist and hip circumference were measured using standard methods [31]. BMI was calculated as weight (kg)/height (m^2^) and standard weight classifications were employed—underweight (< 18.5 kg/m^2^), healthy weight (18.5–24.9 kg/m^2^), overweight (25.0–29.9 kg/m^2^) and obese (≥ 30.0 kg/m^2^) [32]. Body composition was measured by whole-body scan using DEXA (Lunar iDXA, GE Healthcare, Australia) [33].

### High and low salt test meals

Modified versions of published tomato soup formulations [24, 25] were used. The low-salt soup contained 25 g of concentrated tomato (Leggo’s Tomato Paste No Added Salt; Simplot Australia Pty Ltd; 0.02 g salt; 9 mg sodium) dissolved in 250 mL water. The high-salt soup had an identical formulation with 3.80 g of table salt added (final formulation = 3.82 g of salt; 1529 mg sodium). All other nutrients were identical between the test meals (energy = 82 kJ; protein = 0.9 g; carbohydrate = 3.25 g; fat = 0.15 g; potassium = 218 mg).

### Sampling—saliva, blood, and urine

Saliva samples were collected using Salivettes (Sardstedt, Germany). Blood samples (10 mL) were collected via an intra-venous cannula placed in an antecubital vein of the arm. Saliva and blood samples were centrifuged (Heraeus Megafuge 8R Centrifuge, Thermo Fisher Scientific, USA) at 3110 RCF for 10 min and the supernatant stored at − 80 °C [26]. For urine samples, participants visited the bathroom and collected complete urine samples. Total urine volume for each sample was measured and aliquots (2 mL) stored at − 80 °C.

### HR, SBP and DBP

Seated HR, SBP and DBP were measured using an automated clinical sphygmomanometer (Criticare systems Inc, Wisconsin, USA).

### Assays

Urinary sodium was measured by a commercial pathology laboratory (Dorevitch Pathology, Australia) using indirect ion-selective electrode potentiometry (A-LYTE Integrated Multisensor (IMT Na K Cl); Siemens Healthcare Diagnostics Inc, NY, USA).

Enzyme-linked immunosorbent assays (ELISAs) were used to analyze urinary cortisol (DKO018, Diametra, Spello, Perugia, Italy), salivary cortisol (DKO020, Diametra, Spello, Perugia, Italy) and plasma ACTH (RE53081, IBL International GmbH, Hamburg, Germany) in accordance with the manufacturer’s instructions. Samples were assayed in duplicate. Intra-assay variation for these assays was ≤ 8.1% for urinary cortisol, ≤ 10% for salivary cortisol and ≤ 10.3% for plasma ACTH. Inter-assay variation was ≤ 12% for urinary cortisol, ≤ 8.3% for salivary cortisol and ≤ 7.1% for plasma ACTH.

Blood glucose concentrations were measured in a drop of blood using a blood glucose meter (Accu-chek Performa, Roche Diagnostics, Switzerland) prior to centrifugation of each blood sample.

### Statistical analysis and sample size

Descriptive characteristics are reported as mean ± SD or percentage. Statistical analyses were performed using SPSS 27 (SPSS Inc, Chicago, USA). The accepted level of significance was p < 0.05. Prior to analysis, urinary sodium and urinary cortisol for each sample were adjusted for urine volume for the respective sample. Due to technical difficulties, two saliva samples were missing for one participant (T = − 20 and 10 min). To mitigate against exclusion of all data for this participant from time-series analysis, these values were replaced using a conservative approach—by taking the mean of one value before and one value after each missing value. Normality of data was checked using Kolmogorov–Smirnov tests and, where appropriate, data were transformed using the most conservative approach in each case—log_10_ transformation for urinary sodium, urinary and salivary cortisol, HR and DBP and square root transformation for plasma ACTH. To determine the response to high and low salt meals, repeated measures analysis of variance (ANOVA) was conducted with between subject factors of sex (male or female) and order of treatment (high salt first or high salt second) and within subject factors of treatment (high salt meal and low salt meal) and time (see below). For urine measures, 10 time-points were included (Time = 0, 20, 40, 60, 80, 100, 120, 140, 160, 180 min). Since additional measurements were made for saliva, plasma, HR, SBP and DBP measures (Time = 10, 30, 50 min), 13 time points were included for these variables. The sample collected at Time = 0 min was collected immediately before the test meal and was used as the pre-treatment/baseline value. While the earlier pre-treatment values (Time = − 60, − 40, − 20 min) were valuable in illustrating biomarkers are highly variable at the beginning of an experimental study and take time to stabilize, these values were excluded from time-series analyses to enable testing of a priori hypotheses focused on the response to the test meals. Significant interactions were investigated by considering each condition separately and subsequent main effects of time were investigated using within subject post-hoc analysis by Fishers least significant differences. Time-series data are presented as mean ± standard error of the mean (mean ± SEM). Integrated responses (from Time = 0 min onwards) were investigated following calculations of area under the curve with respect to ground (AUCg) and with respect to increase (AUCi) using the trapezoid method [34]. Transformed data were used where applicable. For calculations of AUC_*i*_, the value at time = 0 min was first subtracted from all values. Paired T-tests compared AUCg and AUCi following the high salt meal with that following the low salt meal.

This was a proof-of-concept study with sample size based on previous studies published in this field considering the role of salt intake in cortisol production [9, 13]. Post hoc analysis of observed statistical power indicated adequate statistical power for a priori hypotheses focused on the response to the high and low salt test meals. With the current sample size and observed effect sizes (described in results section for each variable), the observed statistical power was 95% for urinary sodium, 80% for urinary cortisol, 90% for salivary cortisol and 89% for plasma ACTH.

## Results

### Participant characteristics

Participant characteristics are shown in Table 1. Data were collected from 4 females and 4 males with an age range of 21–48 years. Mean BMI fell within a healthy range of 18.5–24.9 kg/m^2^ [32]. Mean cardiometabolic risk biomarker levels fell within normal clinical reference ranges.

**Table 1:** Participant characteristics (n = 8) including demographic, anthropometric and cardiometabolic measures

Characteristic	Mean ± SD or percentage
Age (years)	30.5 ± 9.8
Female (%)	50
BMI (kg/m^2^)	22.8 ± 1.3
Total body fat (%)	28.8 ± 5.6
Trunk body fat (%)	29.7 ± 7.8
Waist circumference (cm)	81.1 ± 5.1
Hip circumference (cm)	100.4 ± 4.6
WHR	0.80 ± 0.05
Resting HR (bpm)	70 ± 9
Resting SBP (mmHg)	108 ± 12
Resting DBP (mmHg)	70 ± 7
CRP (mg/L)^a^	1.3 ± 0.7
Triglycerides (mmol/L)	0.8 ± 0.4
Total cholesterol (mmol/L)	4.9 ± 1.3
HDL-cholesterol (mmol/L)	1.5 ± 0.3
Non-HDL-cholesterol (mmol/L)	3.3 ± 1.2
LDL-cholesterol (mmol/L)	2.9 ± 1.1
Cholesterol/HDL ratio	3.2 ± 0.9
Fasting glucose (mmol/L)	4.7 ± 0.1
Insulin (mIU/L)	5.2 ± 3.1

*BMI* body mass index, *WHR* waist to hip ratio, *HR* heart rate, *SBP* systolic blood pressure, *DBP* diastolic blood pressure, *CRP* C-reactive protein

^a^Due to low values, minimum detection threshold was used for all males and two females

### Urinary sodium

**Fig. 2:**
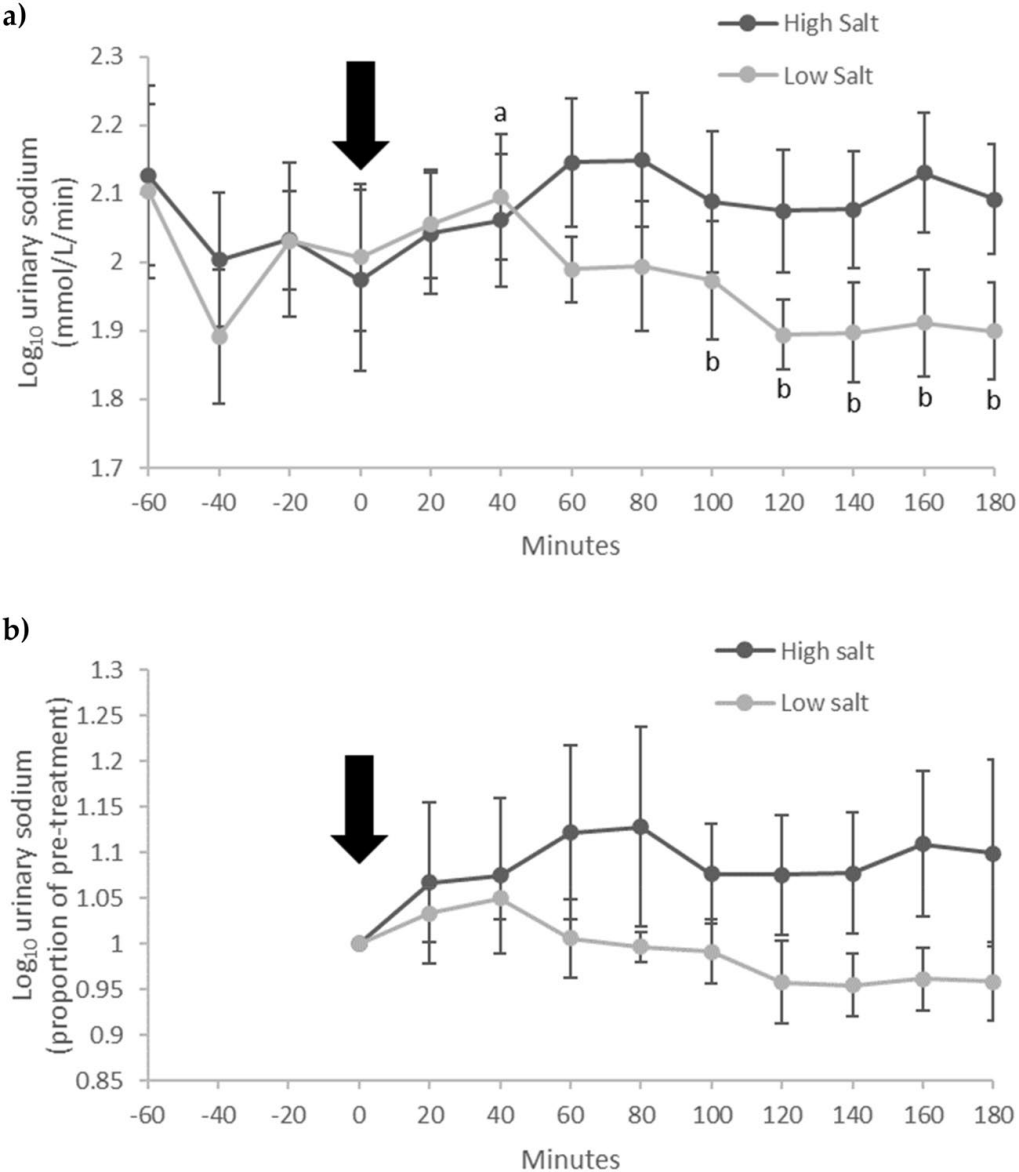
Urinary sodium in participants (n = 8) who consumed high and low salt test meals in a crossover design. Log_10_ urinary sodium data are shown in **a** (treatment*time, F[9, 36] = 3.264, p = 0.005, partial η^2^ = 0.449). For illustrative purposes, **b** shows data as a proportion of pre-treatment. The timing of the test meals is indicated by the arrow. Data are mean (± SEM). ^a,b^Indicates p < 0.05 within the low salt condition

Urinary sodium showed a significant treatment*time interaction (treatment*time, F[9, 36] = 3.264, p = 0.005, partial η^2^ = 0.449) indicating the response to the test meal differed between high and low salt (Fig. 2a). Urinary sodium remained stable following high salt (time effect, F[9, 36] = 1.393, p = 0.228, partial η^2^ = 0.258). In contrast, urinary sodium changed significantly following low salt (time effect, F[9, 36] = 4.229, p = 0.001, partial η^2^ = 0.514) – urinary sodium was highest (4.4% above baseline) 40 min after low salt and was significantly (p < 0.05) lower compared with the peak 100–180 min after the meal (Fig. 2a). The changes in urinary sodium following low but not high salt can be better visualized when the data are plotted as a proportion of pre-treatment (Fig. 2b).

Urinary sodium AUCg did not differ significantly (p = 0.066) following high salt (376.0 ± 15.6) compared with low salt (355.3 ± 12.9). Urinary sodium AUCi was significantly higher (p = 0.046) following high salt (20.7 ± 16.6) compared with low salt (− 6.1 ± 8.7).

### Urinary cortisol

**Fig. 3:**
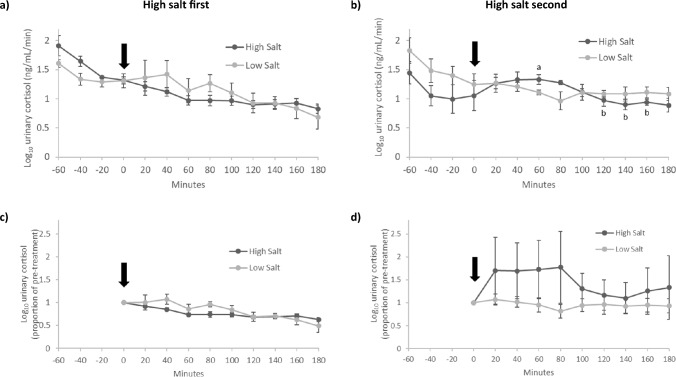
Urinary cortisol in participants who consumed high and low salt test meals in a crossover design. Due to a significant effect of order (treatment*time*order, F[9, 36] = 3.792, p = 0.002, partial η^2^ = 0.487), separate figures are shown for those who had high salt first (**a** and **c**; n = 4) and high salt second (**b** and **d**; n = 4). When high salt was second, there was a significant log_10_ urinary cortisol response (treatment*time, F[9, 18] = 2.641, p = 0.038, partial η^2^ = 0.569; **b**, but not when high salt was first (treatment*time, (F[9, 18]) = 1.517, p = 0.216, partial η^2^ = 0.431; **a**. For illustrative purposes, **c** and **d** show data as a proportion of pre-treatment. The timing of the test meals is indicated by the arrow. Data are mean (± SEM). ^a,b^Indicates p < 0.05 within the high salt condition

Initial analysis of urinary cortisol revealed an effect of treatment order (treatment*time*order, F[9, 36] = 3.792, p = 0.002, partial η^2^ = 0.487), indicating the response to the test meals differed between those who had high salt first and high salt second. Consequently, urinary cortisol data are shown separately for those who had high salt first (n = 4; Fig. 3a) and high salt second (n = 4; Fig. 3b).

High salt first (Fig. 3a, c)—When high salt was first, there was no significant effect of high or low salt on urinary cortisol (treatment*time, (F[9, 18]) = 1.517, p = 0.216, partial η^2^ = 0.431) indicating there was no difference in response between the two meals.

High salt second (Fig. 3b)—When high salt was second, a significant treatment*time interaction was found (treatment*time, F[9, 18] = 2.641, p = 0.038, partial η^2^ = 0.569) indicating the urinary cortisol response was significantly different between high and low salt. Urinary cortisol changed significantly following high salt (time effect, F[9, 18] = 2.795, p = 0.030, partial η^2^ = 0.583) – urinary cortisol was highest (26.3% above baseline) 60 min after high salt and was significantly (p < 0.05) lower compared with the peak from 120 to 160 min after the meal (Fig. 3b). There was no significant change in urinary cortisol following low salt (time effect, F[9, 18] = 1.159, p = 0.375, partial η^2^ = 0.367). The changes in urinary cortisol following high but not low salt can be better visualized when the data are plotted as a proportion of pre-treatment (Fig. 3d).

Urinary cortisol AUCg did not differ significantly following high and low salt either when high salt was first (181.1 ± 11.9 vs 199.6 ± 30.2, respectively, p = 0.424) or second (202.3 ± 14.9 vs 201.9 ± 14.5, respectively, p = 0.985). Urinary cortisol AUCi did not differ significantly following high and low salt either when high salt was first (-55.9 ± 7.4 vs − 36.3 ± 14.2, respectively, p = 0.323) or second (12.2 ± 33.1 vs -22.5 ± 24.9, respectively, p = 0.151).

### Salivary cortisol

**Fig. 4:**
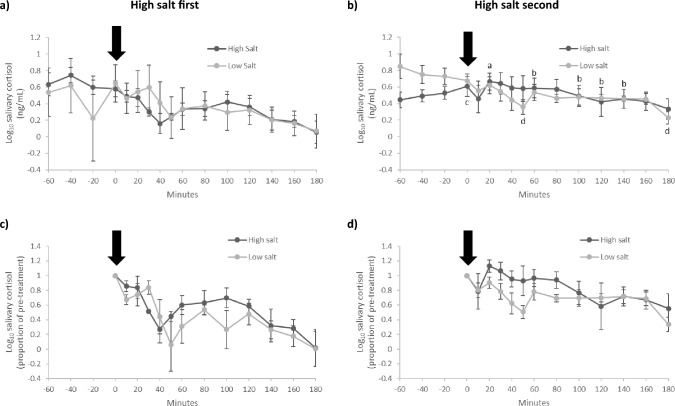
Salivary cortisol in participants who consumed high and low salt test meals in a crossover design. Due to a significant effect of order (treatment*time*order, F[12, 48] = 2.118, p = 0.033, partial η^2^ = 0.346), separate figures are shown for those who had high salt first (**a** and **c**; n = 4) and high salt second (**b** and **d**; n = 4). When high salt was second, there was a significant response in log_10_ salivary cortisol (treatment*time, F[12, 24] = 2.668, p = 0.020, partial η^2^ = 0.572; **b**, but not when high salt was first (treatment*time, F[12, 24] = 1.213, p = 0.330, partial η^2^ = 0.377; **a**. For illustrative purposes, **c** and **d** show data as a proportion of pre-treatment. The timing of the test meals is indicated by the arrow. Data are mean (± SEM). ^a,b^Indicates p < 0.05 within the high salt condition; ^c,d^indicates p < 0.05 within the low salt condition

Initial analysis of salivary cortisol revealed an effect of treatment order (treatment*time*order, F[12, 48] = 2.118, p = 0.033, partial η^2^ = 0.346), indicating the response differed depending on the order of test meals given. Consequently, salivary cortisol data are shown separately for those who had high salt first (n = 4; Fig. 4a) and high salt second (n = 4; Fig. 4b).

High salt first (Fig. 4a, c)—When high salt was first, there was no significant effect of high or low salt on salivary cortisol (treatment*time, F[12, 24] = 1.213, p = 0.330, partial η^2^ = 0.377), indicating there was no difference in response between the meals.

High salt second (Fig. 4b, d)—When high salt was second, there was a significant treatment*time interaction (treatment*time, F[12, 24] = 2.668, p = 0.020, partial η^2^ = 0.572) indicating a significantly different response between high and low salt. Salivary cortisol changed significantly following high salt (time effect, F[12, 24] = 3.021, p = 0.010, partial η^2^ = 0.602)—salivary cortisol was highest (9.4% above baseline) 20 min after high salt and was significantly (p < 0.05) lower compared with the peak 60 min and from 100 to 140 min after high salt (Fig. 4b). There was also a significant change in salivary cortisol following low salt (time effect, F[12, 24] = 6.040, p < 0.001, partial η^2^ = 0.751) – while salivary cortisol did not increase above baseline following low salt, there was a significant (p < 0.05) decrease from baseline (Time = 0 min) to the 50 and 180 min marks after low salt (Fig. 4b). The changes in salivary cortisol following high and low salt can be better visualized when the data are plotted as a proportion of pre-treatment (Fig. 4d).

Salivary cortisol AUCg did not differ significantly following high and low salt either when high salt was first (55.3 ± 15.8 vs 58.7 ± 34.6, respectively, p = 0.882) or second (91.9 ± 21.2 vs 85.2 ± 12.0, respectively, p = 0.662). Salivary cortisol AUCi did not differ significantly following high and low salt either when high salt was first (-48.8 ± 13.8 vs -57.8 ± 10.3, respectively, p = 0.682) or second (-17.6 ± 12.2 vs -36.3 ± 8.7, respectively, p = 0.402).

### Plasma ACTH

**Fig. 5:**
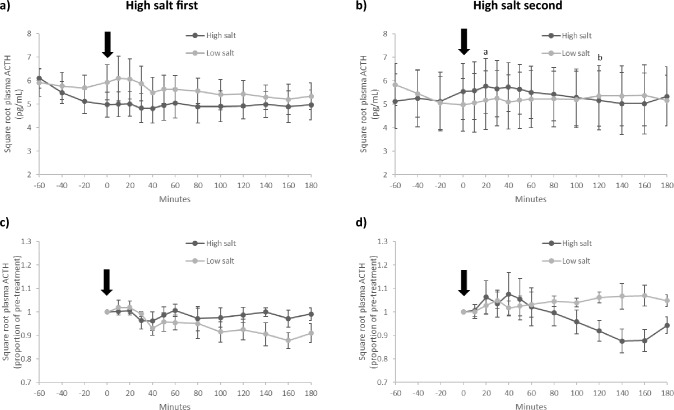
Plasma ACTH in participants who consumed high and low salt test meals in a crossover design. Due to a significant effect of order (treatment*time*order, F[12, 48] = 3.431, p = 0.001, partial η^2^ = 0.462), separate figures are shown for those who had high salt first (**a** and **c**; n = 4) and high salt second (**b** and **d**; n = 4). When high salt was second, there was a significant response in square root plasma ACTH (treatment*time, F[12, 24] = 2.580, p = 0.023, partial η^2^ = 0.563; **b**, but not when high salt was first (treatment*time, F[12, 24] = 1.398, p = 0.234, partial η^2^ = 0.411; **a**. For illustrative purposes, **c** and **d** show data as a proportion of pre-treatment. The timing of the test meals is indicated by the arrow. Data are mean (± SEM). ^a,b^Indicates p < 0.05 within the high salt condition

Initial analysis of plasma ACTH revealed an effect of treatment order (treatment*time*order, F[12, 48] = 3.431, p = 0.001, partial η^2^ = 0.462), indicating the response differed depending on the order of test meals given. Consequently, plasma ACTH data are shown separately for those who had high salt first (n = 4; Fig. 5a) and high salt second (n = 4; Fig. 5b).

High salt first (Fig. 5a, c)—When high salt was first, there was no significant effect of high or low salt on plasma ACTH (treatment*time, F[12, 24] = 1.398, p = 0.234, partial η^2^ = 0.411; 5a), indicating there was no difference in response between the two test meals.

High salt second (Fig. 5b, d)—When high salt was second, there was a significant treatment*time interaction (treatment*time, F[12, 24] = 2.580, p = 0.023, partial η^2^ = 0.563; 5b) indicating the plasma ACTH response was significantly different between high and low salt. Plasma ACTH changed significantly following high salt (time effect, F[12, 24] = 3.617, p = 0.004, partial η^2^ = 0.644)—plasma ACTH was highest (4.1% above baseline) 20 min after high salt and was significantly (p < 0.05) lower than the peak 120 min after the meal (Fig. 5b). There was no significant change in plasma ACTH following low salt (time effect, F[12, 24]) = 0.870, p = 0.585, partial η^2^ = 0.303). The changes in plasma ACTH following high but not low salt can be better visualized when the data are plotted as a proportion of pre-treatment (Fig. 5d).

Plasma ACTH AUCg did not differ significantly following high and low salt either when high salt was first (889.0 ± 110.0 vs 997.5 ± 113.5, respectively, p = 0.212) or second (966.9 ± 216.0 vs 943.7 ± 219.4, respectively, p = 0.514). Plasma ACTH AUCi did not differ significantly following high and low salt either when high salt was first (− 8.2 ± 21.0 vs − 70.3 ± 39.2, respectively, p = 0.359) or second (− 32.0 ± 29.0 vs 46.3 ± 23.4, respectively, p = 0.138).

### HR

HR showed no order effect (treatment*time*order, F[12, 48] = 1.047, p = 0.424, partial η^2^ = 0.207) (Supplementary Fig. 1).

There was no significant difference in HR response following high salt compared with low salt (treatment*time, F[12, 48] = 0.619, p = 0.816, partial η^2^ = 0.134).

HR AUCg did not differ significantly (p = 0.921) following high salt (330.2 ± 3.6) compared with low salt (330.4 ± 4.0). HR AUCi did not differ significantly (p = 0.505) following high salt (− 4.5 ± 1.3) compared with low salt (-3.6 ± 0.8).

### SBP

SBP showed no order effect (treatment*time*order, F[12, 48] = 0.594, p = 0.836, partial η^2^ = 0.129) (Supplementary Fig. 2).

There was no significant difference in the SBP response following high salt compared with low salt (treatment*time, F[12, 48] = 0.800, p = 0.649, partial η^2^ = 0.167).

SBP AUCg did not differ significantly (p = 0.373) following high salt (20,036 ± 892) compared with low salt (19,474 ± 713). SBP AUCi did not differ significantly (p = 0.335) following high salt (258 ± 281) compared with low salt (12 ± 235).

### DBP

DBP showed no order effect (treatment*time*order, F[12, 48] = 0.857, p = 0.594, partial η^2^ = 0.176) (Supplementary Fig. 3).

There was no significant difference in DBP following high salt compared with low salt (treatment*time, F[12, 48] = 0.451, p = 0.933, partial η^2^ = 0.101).

DBP AUCg did not differ significantly (p = 0.937) following high salt (333.3 ± 3.5) compared with low salt (333.5 ± 2.3). DBP AUCi did not differ significantly (p = 0.547) following high salt (2.5 ± 1.0) compared with low salt (1.4 ± 1.5).

### Blood glucose

Blood glucose showed no order effect (treatment*time*order, F[12, 48] = 0.962, p = 0.497, partial η^2^ = 0.194) (Supplementary Fig. 4).

There was no significant difference in the blood glucose response following high salt compared with low salt (treatment*time, F[12, 48] = 1.092, p = 0.388, partial η^2^ = 0.214).

Blood glucose AUCg did not differ significantly (p = 0.966) following high salt (835.6 ± 35.4) compared with low salt (834.7 ± 29.5). Blood glucose AUCi did not differ significantly (p = 0.773) following high salt (− 43.1 ± 21.9) compared with low salt (− 29.3 ± 37.8).

## Discussion

We used a multidisciplinary approach combining experimental methods from nutrition research and stress/psychoneuroendocrinology research to test the first part of our novel working hypothesis—that high salt intake drives cortisol production. Specifically, in our pilot study, we hypothesized that urinary cortisol but not plasma ACTH would increase following a single high-salt meal. In partial support for our hypothesis, we found a peak above baseline following the high salt meal for urinary cortisol (26.3%) and salivary cortisol (9.4%) followed by a significant decline in concentrations in each case when high salt was the second test meal but not the first. Counter to our hypothesis, we also found a modest peak for plasma ACTH (4.1%) above baseline following the high salt meal followed by a significant decline in concentrations when high salt was second but not first. The consistency of findings across three variables (urinary cortisol, salivary cortisol, and plasma ACTH) is noteworthy and deserves discussion and interpretation.

The observed response in urinary cortisol concentrations following the high salt meal (when high salt was given second) occurred in parallel with a response in salivary cortisol levels (when high salt was given second) providing strength to these cortisol outcomes. This is the first time a cortisol response has been demonstrated following a single high salt meal in an acute timeframe. Our urinary cortisol findings align with feeding studies showing 24-h urinary cortisol levels are elevated following 5–7 days of dietary salt-loading [9–12] and provide further support for a direct link between salt intake and cortisol production. The salivary cortisol finding is novel and further supports the link between salt intake and cortisol production. As a steroid hormone, cortisol moves freely from circulating blood into saliva, thus, cortisol found in saliva reflects the free fraction (i.e. that fraction not attached to a binding protein) of circulating cortisol [35, 36]. Indeed, salivary cortisol represents the biologically active fraction of circulating cortisol and is a more sensitive measure of dynamic cortisol activity compared with plasma/serum cortisol [36], in which the presence of binding proteins can complicate the interpretation of dynamic cortisol assessment [37]. These findings support a role for salt intake in the production of cortisol. The observed decline in salivary cortisol from baseline levels following the low salt meal likely reflects the diurnal decline in cortisol known to occur across the day [30].

Interestingly, a cortisol response to salt intake has not previously been captured through measurement of plasma/serum cortisol [7, 15]. Methodological differences may explain these discrepancies since previous studies collected single samples of blood at 8–9 am after 12 h of fasting [7, 15] rather than investigating dynamic cortisol response to a single high-salt meal, as we have done here. In effect, the reason a link between high salt intake and cortisol levels has not previously been seen in plasma/serum cortisol may be that the cortisol response is transient, and the previous sampling protocols [7, 15] did not target the period of response. Based on the current study, the transient cortisol response is complete within around 120–180 min after intake of a high salt meal. In other words, while spot sampling of fasting plasma/serum fails to capture the dynamic response in cortisol, 24-h urine collections capture retrospective evidence of cortisol production since these collections integrate cortisol dynamics across the preceding 24 h. Future studies could test for dynamic plasma/serum cortisol responses, which would help shed light on these discrepancies.

A modest response in plasma ACTH was found, followed by a clear yet delayed decline in plasma ACTH (when high salt was given second). Despite the initial modest ACTH response, the delayed decline in ACTH is the clearest ACTH finding and provides further strength to the cortisol findings by acting as a bioassay. The clear decline in plasma ACTH reflects negative feedback of elevated cortisol on activity of the HPA axis. This delayed decrease in ACTH indicates a physiologically relevant increase in systemic cortisol. The question of the source of salt-induced cortisol production—HPA axis vs visceral adipose production—remains difficult to answer based on our findings. If the HPA axis was responsible for an increase in cortisol following high salt intake, an immediate increase in plasma ACTH following the high salt meal would be expected. The modest ACTH response observed in our study may suggest an involvement of the HPA axis in salt-induced cortisol production, but further research would be needed to confirm this. If the HPA axis is activated by a high salt meal, this may indicate that the body is reacting with a stress response to this stimulus. While the relationship between chronic high salt intake and stress has garnered interest in both human [38] and animal [39] studies, the acute impact of a single high-salt meal on activation of the HPA axis deserves further attention. The delayed decrease is the clearest ACTH change in our study following the high salt meal, but indicates cortisol negative feedback rather than being informative about the source of cortisol production per se. The dynamics of ACTH levels following a high-salt meal warrant further investigation to better understand the role of the HPA axis.

Next, let us consider the order effect whereby significant findings in urinary and salivary cortisol and plasma ACTH were only observed when high salt was given second, but not first. It can be considered that changes in cortisol and ACTH were only seen on moving from low to high salt intake conditions, rather than from ambient to high. While the reasons for this anomaly are not clear, one possible explanation may be the novelty of the testing environment and procedures on the first day of testing—this unfamiliarity and uncertainty may have acted as a very mild stressor leading to low level background cortisol production and, therefore, cortisol negative feedback, which may have limited further production of cortisol. By the second day of testing, participants were familiar with the testing environment and procedures, potentially eliminating any background cortisol negative feedback. An alternative explanation for this anomaly may be that there were differences between participants in ‘salt sensitivity of blood pressure’. Salt sensitivity of blood pressure can be defined as a physiological phenotype in which changes in blood pressure parallel changes in salt intake [40]—resting blood pressure increases as dietary salt intake increases and decreases with dietary salt restriction. Salt sensitivity of blood pressure affects 26% of normotensive individuals [41] and is known to have a genetic component [40]. Salt sensitivity of blood pressure was shown to be related to 24-h urinary free cortisol during dietary salt loading—those with the lowest salt sensitivity had the highest urinary free cortisol—although, caution is warranted in extrapolating these findings since predominantly hypertensive rather than normotensive individuals were investigated [42]. Supporting evidence from normotensive individuals shows increased 24-h urinary free cortisol following dietary salt loading only in individuals who had low salt sensitivity [10]. Perhaps by chance in the current study, there were differences between participants in salt sensitivity of blood pressure which may explain the different outcomes observed between high salt first and high salt second. We did not have the capacity or resources to test salt sensitivity of blood pressure in our participants, which involves multi-day dietary interventions or acute inpatient protocols [40]. Nevertheless, consideration of salt sensitivity of blood pressure would be a valuable addition in future research.

To further characterize the role of high salt intake in an acute setting, we also measured the dynamics of HR, SBP, DBP and blood glucose but we found no differences between the high and low salt meals. These outcomes do not help explain the findings of Schutten et al. that, after a 7-day high salt diet, 24-h SBP and DBP were significantly higher and fasting plasma glucose significantly lower compared with a low salt diet [12]. No difference was found in 24-h HR following 7-days of high and low salt diets [12]. In contrast to our urinary and salivary cortisol findings reported here, the long-term changes in cardiovascular and glucose parameters reported elsewhere [12] do not appear to mirror dynamic changes in response to a single high salt meal suggesting different regulatory processes may be at play across these different physiological systems. Indeed, changes in cortisol may represent a primary outcome of high salt intake whereas cardiovascular and glucose changes may represent secondary outcomes.

There are many strengths of this research. Firstly, the controlled experimental environment allowed for proper testing of our hypothesis in the absence of known confounders. For example, by standardizing the time of day of testing, we mitigated the influence of diurnal changes in cortisol [30] and by excluding participants with disease states and conditions known to influence the HPA axis, we eliminated the potential influence of these factors. By providing a standardized meal on arrival, 2 h before the test meal, we avoided the influence of unknown food intake prior to arrival at the research facility on cortisol levels. Our observed urinary sodium findings suggest there may have been some residual influence of the standardized lunch on urinary sodium during the initial stages of the testing period, but importantly, urinary sodium declined following the low salt meal while remaining stable following the high salt meal allowing us to test our hypothesis appropriately. Limitations of this research are the low sample size and the lack of measurement of salt sensitivity of blood pressure in the study population. Future research is warranted that can address these limitations as well as the longitudinal impact of dietary salt intake on obesity.

## Conclusions

In conclusion, our innovative multidisciplinary approach bringing together methods from disparate disciplines has introduced a novel pathway with potential importance in the development of obesity. The observed responses in urinary and salivary cortisol and plasma ACTH following a single high salt meal are highly intriguing and novel. These findings would benefit from replication and confirmation in further research. Our findings provide impetus for further investigation of our novel working hypothesis that high salt intake drives cortisol production, and in turn, increased cortisol production drives obesity. Such research is expected to uncover cortisol as a novel mechanism linking high salt intake with the development of obesity. In the efforts of clinical and public health practitioners and researchers to understand and address global obesity and its related morbidity and mortality, this line of research may provide a novel target for intervention.

### Supplementary Information

Below is the link to the electronic supplementary material.

Supplementary file1 (DOCX 136 KB)

## Data Availability

The data presented in this study are available upon reasonable request from the corresponding author. The data are not publicly available due to ethical and privacy reasons.
