# Supplementary material for: Is high salt intake inducing obesity via production of cortisol? A novel working hypothesis and pilot study
Source: Eur J Nutr. 2024 Feb 26;63(4):1315–27. doi: 10.1007/s00394-024-03354-6 (PMC11139711; doi:10.1007/s00394-024-03354-6)
Supplement: Supplementary file 1 — Supplementary file1 (DOCX 136 KB) [file 394_2024_3354_MOESM1_ESM.docx]

***European Journal of Nutrition***

**Is high salt intake inducing obesity via production of cortisol? – A novel working hypothesis and pilot study**

Anthony Nowell ^1^, Susan J. Torres ^1^, Sarah J. Hall ^1^, Michelle A. Keske ^1^, David J. Torpy ^2^, Lewan Parker ^1^, Andrew C. Betik ^1^, and Anne I. Turner ^1,^*

^1^ Institute for Physical Activity and Nutrition, Deakin University, Victoria, Australia

^2^ Endocrine and Metabolic Unit, Royal Adelaide Hospital, South Australia, Australia

***** Correspondence: [anne.turner@deakin.edu.au](mailto:anne.turner@deakin.edu.au)

**Supplementary Figures**

| **a)**  **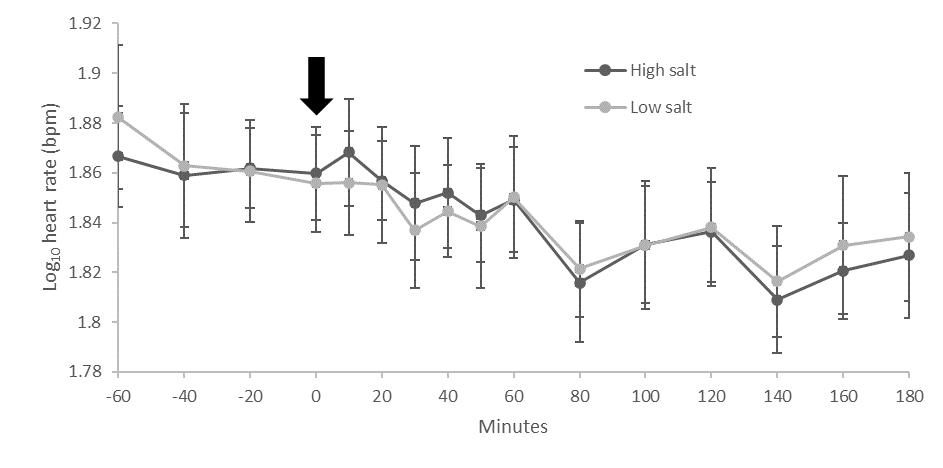** |
| --- |
| **b)**  **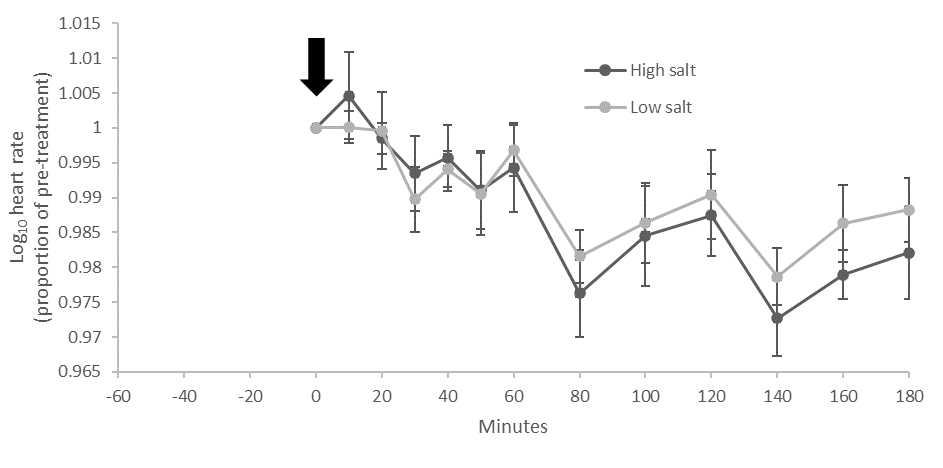** |

**Supplementary Fig 1** HR in participants (n = 8) who consumed high and low salt test meals in a crossover design. Log_10_ HR data are shown in 1a (treatment*time, F[12, 48] = 0.619, p = 0.816, partial η^2^ = 0.134). For illustrative purposes, 1b shows data as a proportion of pre-treatment. The timing of the test meals is indicated by the arrow. Data are mean (±SEM)

| **a)**  **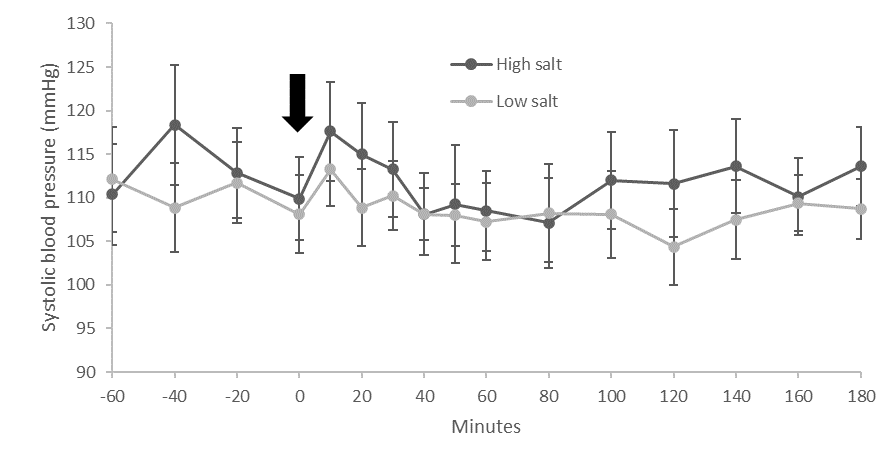** |
| --- |
| **b)**  **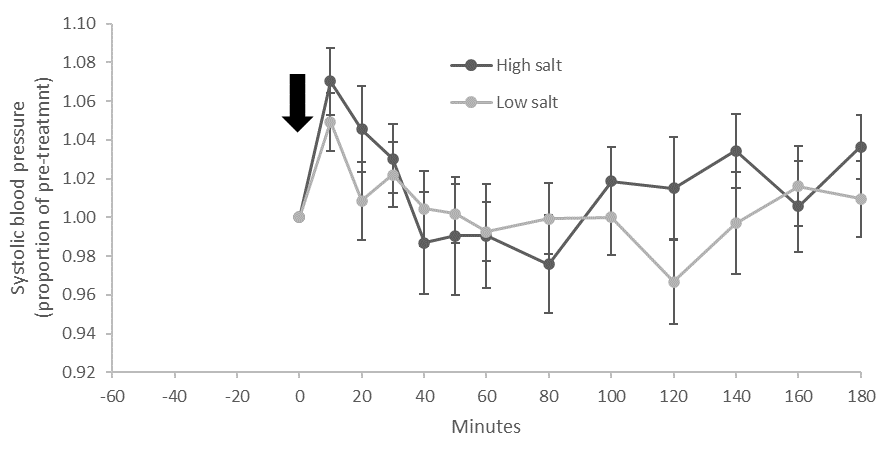** |

**Supplementary Fig 2** SBP in participants (n = 8) who consumed high and low salt test meals in a crossover design. Raw SBP data are shown in 2a (treatment*time, F[12, 48] = 0.800, p = 0.649, partial η^2^ = 0.167). For illustrative purposes, 2b shows data as a proportion of pre-treatment. The timing of the test meals is indicated by the arrow. Data are mean (±SEM)

| **a)**  **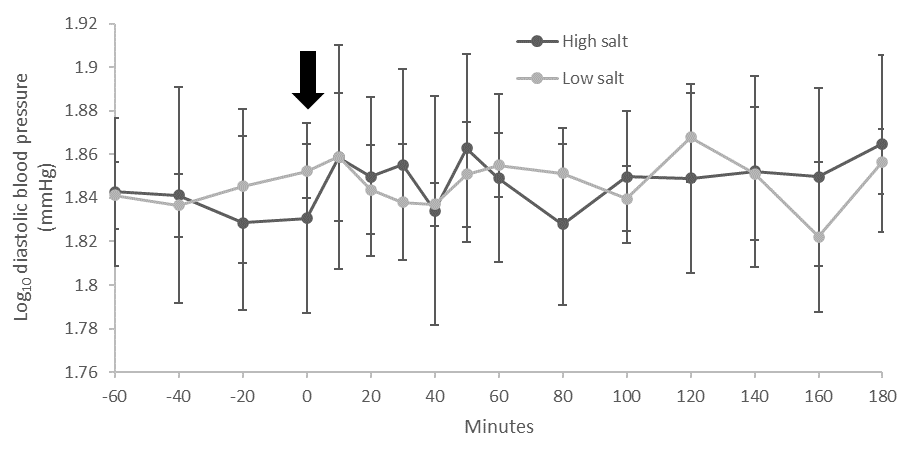** |
| --- |
| **b)**  **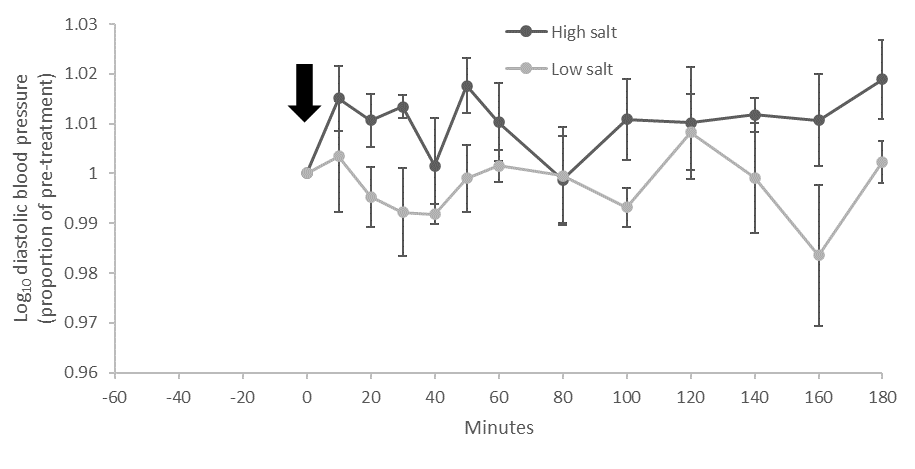** |

**Supplementary Fig 3** DBP in participants (n = 8) who consumed high and low salt test meals in a crossover design. Log_10_ DBP data are shown in 3a (treatment*time, F[12, 48] = 0.451, p = 0.933, partial η^2^ = 0.101). For illustrative purposes, 3b shows data as a proportion of pre-treatment. The timing of the test meals is indicated by the arrow. Data are mean (±SEM)

| **a)**  **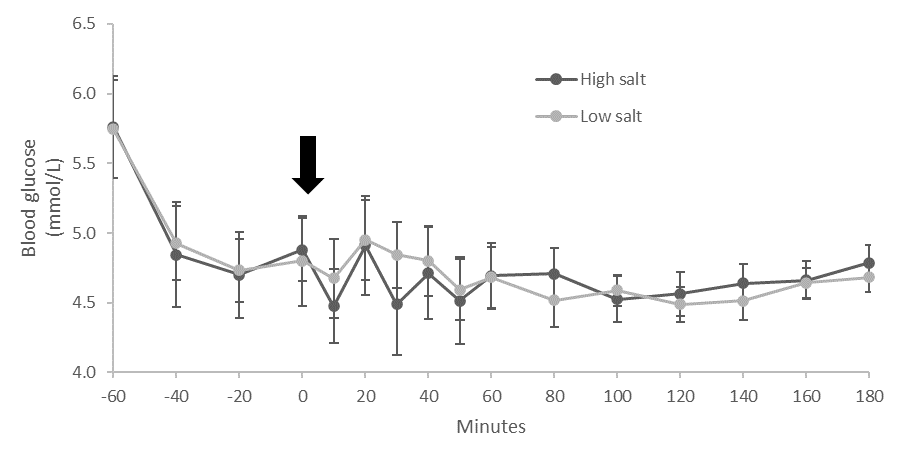** |
| --- |
| **b)**  **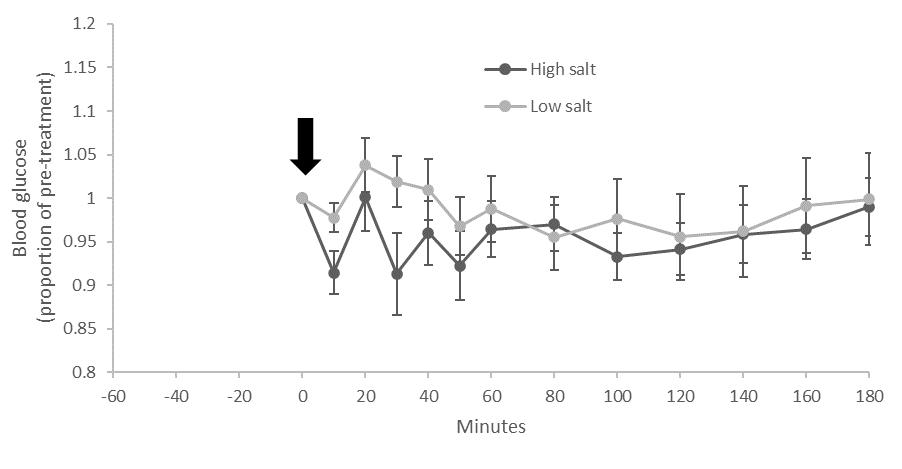** |

**Supplementary Fig 4** Blood glucose in participants (n = 8) who consumed high and low salt test meals in a crossover design. Blood glucose data are shown in 4a (treatment*time, F[12, 48] = 1.092, p = 0.388, partial η^2^ = 0.214). For illustrative purposes, 4b shows data as a proportion of pre-treatment. The timing of the test meals is indicated by the arrow. Data are mean (±SEM)
